# Genetic Mutations of Tim-3 Ligand and Exhausted Tim-3^+^ CD8^+^ T Cells and Survival in Diffuse Large B Cell Lymphoma

**DOI:** 10.1155/2020/6968595

**Published:** 2020-10-29

**Authors:** Tingting Zhang, Tianyuan Ren, Zheng Song, Jing Zhao, Lei Jiao, Zhenzhen Zhang, Jin He, Xianming Liu, Lihua Qiu, Lanfang Li, Shiyong Zhou, Bin Meng, Qiongli Zhai, Xiubao Ren, Zhengzi Qian, Xianhuo Wang, Huilai Zhang

**Affiliations:** ^1^Department of Lymphoma, Tianjin Medical University Cancer Institute and Hospital, National Clinical Research Center of Cancer, Key Laboratory of Cancer Prevention and Therapy, Tianjin's Clinical Research Center for Cancer, The Sino-US Center for Lymphoma and Leukemia Research, Tianjin, China; ^2^Panovue Biological Technology Co., Ltd., Beijing, China; ^3^Marvel Medical Laboratory, Tianjin Marvelbio Technology Co. Ltd., Tianjin, China; ^4^Department of Pathology, Tianjin Medical University Cancer Institute and Hospital, Tianjin, China; ^5^Department of Immunology/Biotherapy, Tianjin Medical University Cancer Institute and Hospital, Tianjin, China

## Abstract

Tim-3 is a promising target for antitumor immunotherapy. A number of clinical trials are evaluating the efficacy of anti-Tim-3 therapies as a single agent or combinations in solid tumors and haematologic malignancies. However, there remains a considerable lack of data on Tim-3 signalling, especially the genetic characteristics and immune microenvironment, in diffuse large B cell lymphoma (DLBCL). Herein, we identified three genetic mutations in galectin-9, a major ligand of Tim-3, in six patients with DLBCL (6/188, 3.2%) that were not detected in the COSMIC database. The Oncomine database showed that the mRNA levels of Tim-3 were higher in DLBCL cells than those in normal B cells. Multiplexed immunofluorescence revealed that patients with Tim-3-expressing tumor-infiltrating lymphocytes (Tim-3^+^ TILs) exhibited poor outcomes than those with Tim-3^−^ TILs (*p* = 0.041). The median survival times of these patients were 65.0 (95% confidence interval (CI): 71.2–88.6) and 79.9 months (95% CI: 54.4–75.6), respectively. Furthermore, we defined a novel subtype of exhausted T cells, named as exhausted Tim-3^+^ CD8^+^ T cells, and found that patients with exhausted Tim-3^+^ CD8^+^ T cells (median survival, 62.8 months, 95% CI: 50.0–75.6) exhibited shorter survival than those with nonexhausted Tim-3^−^ CD8^+^ T cells (median survival, 82.5 months, 95% CI: 72.0–92.9; *p* = 0.034). Overall, these findings provide the genetic status of the Tim-3 ligand in DLBCL. Patients with Tim-3^+^ TILs and exhausted Tim-3^+^ CD8^+^ T cells exhibited inferior survival, thus highlighting the possibility of potential therapeutic applications of the inhibition of Tim-3 alone or in combination with other immune checkpoints for treatment of patients with DLBCL.

## 1. Introduction

Diffuse large B cell lymphoma (DLBCL) is the most common type of non-Hodgkin's lymphoma and is characterized by a highly aggressive and heterogeneous disease course. Although the addition of rituximab to traditional chemotherapy has significantly improved the survival of patients with DLBCL, 30~40% of patients experience relapse and/or refractory disease and have a poor prognosis [1]. Therefore, it is imperative to develop new therapeutic strategies based on the underlying pathological mechanisms of DLBCL.

In recent years, targeting immune checkpoints using PD-1/PD-L1 inhibitors has been promising in treating a variety of tumors, such as melanoma, lung cancer, and Hodgkin's lymphoma [2]. However, except primary mediastinal large B cell lymphoma (a special type of DLBCL), PD-1/PD-L1 therapeutics are not efficacious and a large proportion of patients with DLBCL do not benefit from these therapies [3]. This warrants the need for identifying novel molecules that target immune checkpoints and escape mechanisms [4].

Tim-3, a member of the T cell immunoglobulin and mucin domain family of proteins, was discovered as an inhibitor of Th1 cells and has been reported to maintain immunologic tolerance [5]. Tim-3 signalling has recently been shown to directly regulate the function of CD8^+^ T cells through various mechanisms [6, 7]. In preclinical models of solid and haematologic malignancies, Tim-3 is crucial in mediating T cell exhaustion and generating an immunosuppressive microenvironment [8, 9]. Tim-3 is also expressed in other immune cells and inhibits the activation of antitumor immune response [10]. Targeting Tim-3 signalling has shown promise in multiple tumor models. In colon carcinoma (CT26 and MC38), Tim-3 blockade alone exhibited similar efficacy as blocking the PD-1 pathway [11]. Furthermore, the combination of Tim-3 and PD-1 pathway blockade also showed a remarkable synergistic effect [9]. These observations highlight the importance of Tim-3 as a promising target for antitumor immunotherapy. A large number of clinical trials are underway to test the efficiency of anti-Tim-3 antibodies in different types of tumors [4]. However, the role of Tim-3 signalling in DLBCL, especially genetic mutations and immune microenvironment, remains to be elucidated. PD-1 is a molecule involved as a hallmark for T cell exhaustion [12]. Here, we have defined a novel subtype of exhausted T cells, named exhausted Tim-3^+^ CD8^+^ T cells.

To date, four ligands have been shown to interact with Tim-3, and galectin-9 is identified as one of the major ligands [13]. In this study, we performed targeted deep sequencing to investigate the genetic mutations associated with galectin-9. Microarray data were obtained from the Oncomine database. The expression of Tim-3 in the total tumor-infiltrating lymphocytes (TILs) and CD8^+^ T cells was measured using multiplexed immunofluorescence and digital imaging techniques. Furthermore, we analysed the correlation between Tim-3^+^ TILs, exhausted Tim-3^+^ CD8^+^ T cells, and clinical outcome.

## 2. Materials and Methods

### 2.1. Patients and Samples

Two cohorts were enrolled in this study, including 188 and 134 patients with DLBCL from whom fresh frozen and formalin-fixed paraffin-embedded tumor samples were available for sequencing and immunofluorescence staining, respectively. All the samples were collected at Tianjin Medical University Cancer Institute and Hospital (TMUCIH). Each biopsy was reviewed by two experienced haematopathologists for diagnostic confirmation. The study was approved by the Clinical Research Ethics Board of TMUCIH, and written informed consent was obtained from all patients. This study was conducted in accordance with the Declaration of Helsinki (1964). Tim-3 expression arrays (*n* = 64) were obtained from the Oncomine database to compare the mRNA levels of Tim-3 between tumor and normal tissues.

### 2.2. DNA Extraction

Genomic DNA from fresh tumor biopsies were extracted using the DNeasy Blood & Tissue Kit (Qiagen, Hilden, Germany) according to the manufacturer's recommendations, and DNA quality was checked. A total amount of 0.6–1 *μ*g DNA per sample was used as input material to construct the library.

### 2.3. Targeted Deep Sequencing

We designed a custom panel of 307 genes, including galectin-9. Gene-specific primers were designed to flank the exons of the target genes. Subsequently, we sequenced the 188 tumor specimens using the Illumina Hiseq X10 platform (San Diego, CA, USA). The sequencing data were mapped to the reference human genome (UCSC hg19) using the Burrows-Wheeler Aligner software. Recalibration of reads and variant calling were performed using the Genome Analysis Toolkit. Somatic mutations were identified using MuTect [14].

### 2.4. Multiplexed Immunofluorescence Staining

Multiplexed immunofluorescence staining was performed as described in our previous study [15], to visualise the expression of Tim-3, CD8, and PAX-5. Here, PAX-5 was used to localise the tumor cells. Formalin-fixed paraffin-embedded whole tissue sections were deparaffinised and subjected to antigen retrieval using citrate solution (pH = 6.0). Slides were placed in a microwave for 4 min at 100% power and for an additional 15 min at 20% power. The tissues were blocked with blocking solution (Dako Antibody Diluent) for 10 min and incubated overnight with an anti-PAX-5 primary antibody (1: 2,000, clone: SP34, Roche, Basel, Switzerland) at 4°C. Subsequently, they were incubated with anti-rabbit horseradish peroxidase-conjugated secondary antibody (PerkinElmer) for 10 min at room temperature and labelled using Opal 540 tyramide signal amplification reagents (PerkinElmer) for 10 min. The primary-secondary-horseradish peroxidase complex was removed using a microwave, and the sections were stained for Tim-3/Opal 620 (1: 1,000, clone: D5D5R, Cell Signaling Technology, MA, USA) and CD8/Opal 520 (1: 1,000, polyclonal, Abcam, Cambridge, UK). After three rounds of staining, the sections were counterstained with DAPI (Life Technologies). Coverslips were mounted on the slides using ProLong Gold antifade with DAPI and stored in a lightproof box at 4°C prior to imaging.

### 2.5. Quantitative Analysis of Tim-3 Expression

Multiplex-stained slides were imaged using the Mantra System (PerkinElmer). Based on PAX-5 staining, we acquired multispectral images (×200 magnification) from 20 random fields of view on each slide. Raw images were analysed using the inForm 2.4.0 software (PerkinElmer) [15]. The absolute cell numbers per mm^2^ that demonstrate marker staining were automatically analysed using a professional computer-assisted platform (PerkinElmer). Briefly, each DAPI-stained cell was identified according to the pattern of fluorophore expression and nuclear/cell morphological features (PAX-5^+^/Opal 540 for tumor cells and CD8^+^/Opal 520 for CD8^+^ TILs). The cell numbers per mm^2^ were calculated according to the algorithm: the addition of cell numbers in fields captured divided by the area of these fields. Median values were used as the cut-off values to distinguish different groups. Tim-3 positivity (Tim-3^+^ TILs) was defined as >median cells/mm^2^ (37 cells/mm^2^), which exhibited membrane staining for Tim-3 with a DAPI-stained component, but not PAX-5 staining. A previous study has reported that the “immune-inflamed phenotype” is characterised by the presence of CD8-expressing T cells in the tumor parenchyma [16]. We precisely defined CD8^+^ T cells > 100 cell numbers/mm^2^ as the “immune-inflamed phenotype.” The role of exhausted T cells was further analysed in patients with this phenotype. Here, exhausted T cells (Tim-3^+^ CD8^+^ T cells) were defined as >median cells/mm^2^ (12 cells/mm^2^), which exhibited membrane staining for Tim-3 and CD8. Nonexhausted T cells (Tim-3^−^ CD8^+^ T cells) were defined as <median cells/mm^2^ (12 cells/mm^2^), which exhibited membrane staining for Tim-3 and CD8.

### 2.6. Statistical Analyses

Quantitative variables of Tim-3 mRNA levels were analysed using Student's *t*-test. The correlation between Tim-3 expression and clinicopathological characteristics was estimated using Pearson's chi-squared test. Progression-free survival (PFS) was defined as the time from the day of diagnosis to the day of relapse, progression, or death from any cause. Overall survival (OS) was defined as the time from the day of diagnosis to the day of death or last follow-up. Kaplan-Meier curves were used to calculate survival. Statistical analyses were performed using SPSS 22.0, and *p* < 0.05 was considered statistically significant.

## 3. Results

### 3.1. Genetic Mutations in the Tim-3 Ligand

**Figure 1:**
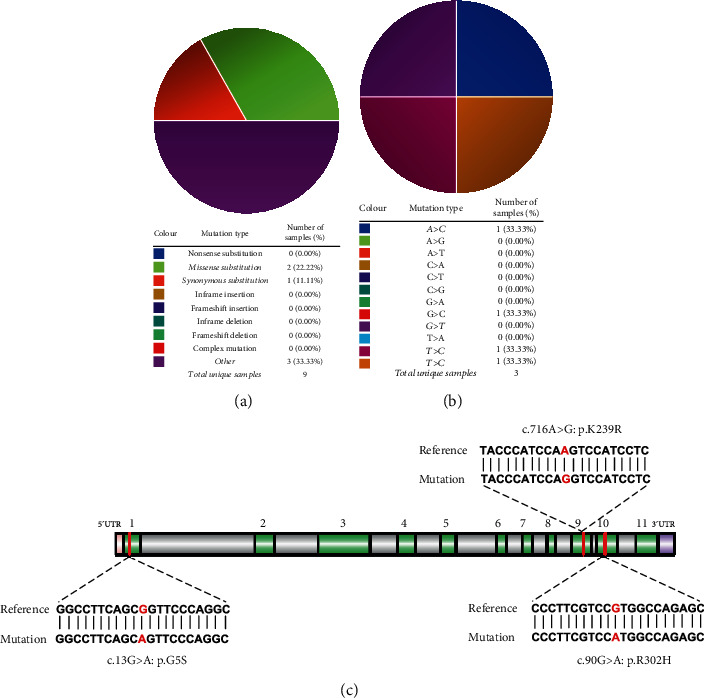
Genetic mutations in galectin-9: (a) an overview of the mutations in galectin-9 in the samples from patients with haematopoietic and lymphoid malignancies, according to the COSMIC database. (b) The substitution mutations of galectin-9 in the haematopoietic and lymphoid cancer samples, according to the COSMIC database. (c) Schematic representation of galectin-9, indicating the location of the mutations identified by targeted deep sequencing in patients with diffuse large B cell lymphoma (DLBCL).

First, we searched for the mutants of galectin-9 using the COSMIC database (http://cancer.sanger.ac.uk/cosmic): there were mutations in 9 of 4,589 (0.2%) patients in haematopoietic and lymphoid cancers. One patient with acute myeloid leukaemia had two missense mutations, namely, c.647 T > G and c.652 T > C. The c.562A > C missense mutation was detected in a patient with mantle cell lymphoma. Similarly, a patient with an unknown disease possessed the c.672G > T synonymous mutation (Figures 1(a) and 1(b)). Furthermore, the database showed three and four patients with mutations in the 5′ untranslated region and introns of galectin-9, respectively.

We performed targeted deep sequencing of galectin-9 using samples from the 188 patients with DLBCL. The patients had a median age of 61 years (range, 17–91 years). Among the patients, 59.0%, 41.0%, and 28.2% were males, had stage III-IV cancer, and had an IPI score of >2, respectively. A total of 80 and 76 patients were subjected to R-CHOP-like and CHOP-like treatment regimens, respectively. The remaining patients were not administered any treatment for personal reasons. Three galectin-9 mutation sites were identified in six cases (6/188, 3.2%; Figure 1(c)). Among them, four patients harboured the same base substitution of c.13G > A that replaces the glycine with serine at position 5 of galectin-9 (p.G5S). Furthermore, one patient possessed the c.905G > A mutation that alters the arginine at the 302^nd^ position to histidine (p.R302H). Notably, this mutation was predicted by the SIFT (score 0.01) and Polyphen2 (score 0.971) algorithms to be deleterious and damaging, suggesting altered protein function. Another missense mutation detected was c.716A > G that was responsible for mutating lysine at the 239^th^ position in galectin-9 to arginine (p.K239R). Importantly, these mutations were not detected in the DLBCL sample part of the COSMIC database.

### 3.2. High mRNA Levels of Tim-3 in DLBCL

**Figure 2:**
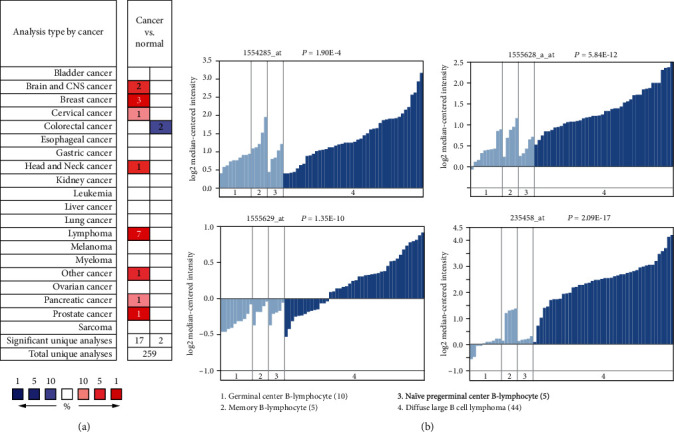
The mRNA levels of Tim-3 in tumor and normal tissues from the Oncomine database: (a) upregulation of Tim-3 was found in 8 of 20 cancer types, including lymphoma. (b) Tim-3 mRNA levels were significantly higher in DLBCL cells than normal B cells, including germinal center B-lymphocytes, memory B-lymphocytes, and naïve pregerminal center B-lymphocytes using four probes (1554285_at, 1555628_a_at, 1555629_at, and 235458_at) with the Compagno Lymphoma dataset.

Next, we studied whether the mRNA levels of Tim-3 in DLBCL were different from those in normal B cells. We compared the microarray data obtained from the Oncomine database (https://www.oncomine.org). Tim-3 was upregulated in 8 of 20 cancer types including lymphoma (Figure 2(a)). Using the Compagno Lymphoma dataset and multiple probes, we found that the mRNA levels of Tim-3 were higher in DLBCL cells as compared to those in normal B cells (*p* = 1.90*E* − 4 for probe 1554285_at, *p* = 5.84*E* − 12 for probe 1555628_a_at, *p* = 1.35*E* − 10 for probe 1555629_at, and *p* = 2.09*E* − 17 for probe 235458_at; Figure 2(b)).

### 3.3. Tim-3 Expression and Clinicopathological Features in DLBCL

**Figure 3:**
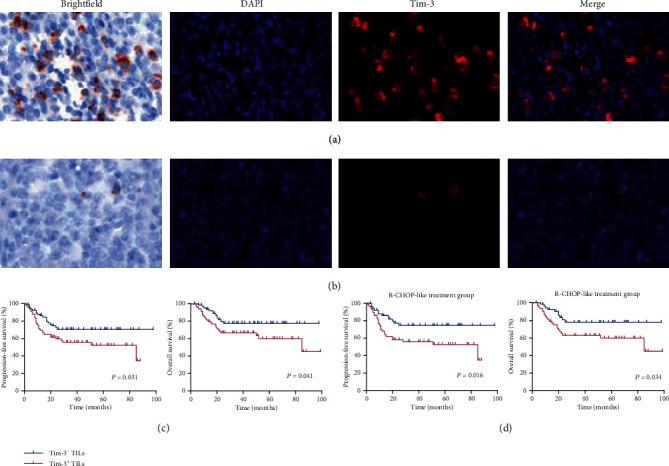
Expression of Tim-3 on tumor-infiltrating lymphocytes (TILs) and survival of patients with DLBCL: (a, b) Representative images of brightfield and fluorescence microscopy for the expression of Tim-3 on TILs. (c) Kaplan-Meier curves for progression-free survival (PFS) and overall survival (OS) in DLBCL patients with Tim-3^+^ TILs and Tim-3^−^ TILs (*n* = 134). (d) Kaplan-Meier curves for PFS and OS in DLBCL patients with Tim-3^+^ TILs and Tim-3^−^ TILs in the R-CHOP-like treatment subgroup (*n* = 109). DAPI (blue) and Tim-3 (red) staining. Scans were imaged at 200x magnification. Log-rank test was used to determine the significance of comparison between two groups.

To explore the clinical significance of Tim-3 protein levels in DLBCL, we performed immunofluorescence of another cohort of 134 patients with DLBCL. Among these formalin-fixed paraffin-embedded samples, 67 were from lymph nodes, and the remaining were from extranodal organs, including gastrointestinal tissues (*n* = 43), thyroid (*n* = 7), soft tissue (*n* = 5), bone (*n* = 3), testis (*n* = 3), breast (*n* = 2), ovary (*n* = 1), kidney (*n* = 1), brain (*n* = 1), and lung (*n* = 1). The median age of these patients was 58 years (range, 22–84 years). Among these patients, 50%, 52.2%, and 26.2% were male, had stage III-IV cancer, and had middle-high or high-risk IPI, respectively. A total of 109 and 25 patients were administered R-CHOP-like and CHOP-like treatment regimens, respectively. The patients were divided into two groups based on the median expression of Tim-3 on TILs, and the association between various clinicopathological factors was analysed. Patients with Tim-3^+^ TILs had a higher frequency of IPI scores of >2 (*p* = 0.036), advanced stages (*p* = 0.038), and high LDH levels (*p* = 0.033) than those in patients with Tim-3^−^ TILs. Patient age, sex, and B symptom and subtype were not significantly different between the two groups (Table 1). Figures 3(a) and 3(b) showed the representative multispectral images of Tim-3^+^ and Tim-3^−^ TILs.

### 3.4. Prognostic Value of Tim-3^+^ TILs and Exhausted Tim-3^+^ CD8^+^ T Cells in DLBCL

We further analysed the correlation between Tim-3^+^ TILs and survival in DLBCL patients. The median follow-up duration was 32 months (range, 3–99 months) for all patients. The median survival times for patients with Tim-3^+^ TILs and Tim-3^−^ TILs were 65.0 (95% confidence interval (CI): 71.2–88.6) and 79.9 months (95% CI: 54.4–75.6), respectively. Patients with Tim-3^+^ TILs exhibited shorter PFS and OS than those with Tim-3^−^ TILs (*p* = 0.031 and *p* = 0.041, respectively; Figure 3(c)). Similar results were observed upon limiting the analysis to patients treated with the R-CHOP-like regimen (*n* = 109, *p* = 0.016 and *p* = 0.034, respectively; Figure 3(d)).

**Figure 4:**
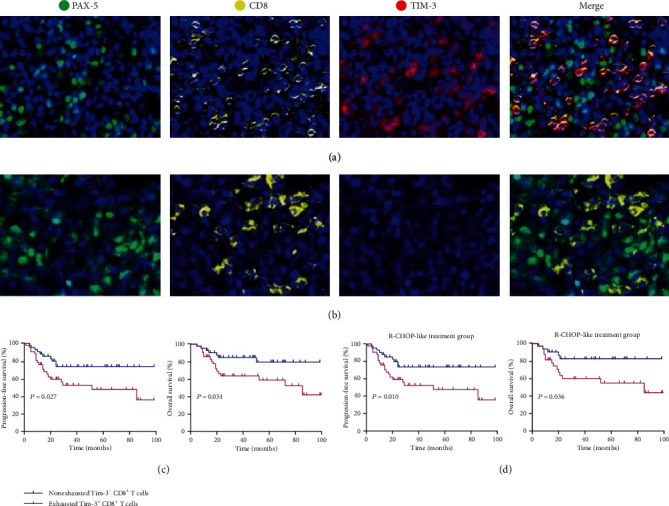
Exhausted Tim-3^+^ CD8^+^ T cells and nonexhausted Tim-3^−^ CD8^+^ T cells and survival of patients with DLBCL. (a, b) Representative fluorescence microscopy images of exhausted Tim-3^−^ CD8^+^ T cells and nonexhausted Tim-3^−^ CD8^+^ T cells. (c) Kaplan-Meier curves for PFS and OS of “immune-inflamed phenotype” patients with DLBCL and exhausted Tim-3^+^ CD8^+^ T cells and nonexhausted Tim-3^−^ CD8^+^ T cells (*n* = 82). (d) Kaplan-Meier curves for PFS and OS of patients with DLBCL and exhausted Tim-3^+^ CD8^+^ T cells and nonexhausted Tim-3^−^ CD8^+^ T cells in the R-CHOP-like treatment subgroup (*n* = 63). DAPI (blue), PAX-5 (green), CD8 (yellow), and Tim-3 (red) were stained. Scans were imaged at 200x magnification. Log-rank test was used to determine the significance of comparison between two groups.

Multiple studies have demonstrated that high CD8^+^ T cell density was associated with an improved prognosis and survival [17, 18]. However, exhausted CD8^+^ T cells are a distinct subtype within cytotoxic CD8^+^ T cells with impaired function. Here, we defined and characterised 82 patients with CD8^+^ T cells > 100 cell numbers/mm^2^ with the “immune-inflamed phenotype.” Figures 4(a) and 4(b) showed the representative multispectral images of exhausted Tim-3^+^ CD8^+^ T cells and nonexhausted Tim-3^−^ CD8^+^ T cells. We analysed the effect of exhausted Tim-3^+^ CD8^+^ T cells on the survival of patients with DLBCL. Patients with exhausted Tim-3^+^ CD8^+^ T cells had inferior PFS and OS compared with those with nonexhausted Tim-3^−^ CD8^+^ cells (*p* = 0.027 and *p* = 0.034, respectively; Figure 4(c)). The median survival times of the patients in the exhausted and nonexhausted groups were 62.8 (95% CI: 50.0–75.6) and 82.5 months (95% CI: 72.0–92.9), respectively. Similar results were also observed in patients treated with the R-CHOP-like regimen (*p* = 0.010 and *p* = 0.036, respectively; Figure 4(d)).

## 4. Discussion

Targeting novel checkpoint receptors for anticancer immunotherapy has increasingly attracted attention among researchers. Several properties of Tim-3 make it an ideal target for the next generation of immunotherapy. CTLA-4 and PD-1 are upregulated on all effector T cells; CTLA-4 is also expressed on all Tregs. Autoimmune-like toxicity is commonly observed in patients treated with anti-CTLA-4 and/or PD-1 antibody [19]. However, Tim-3 is selectively expressed on intratumoral T cells in cancer patients [20, 21], suggesting that targeting Tim-3 signalling reduces nonspecific toxicity. Moreover, Tim-3 affects the downstream signalling pathways of T cell receptor activation different from that regulated by CTLA-4 and PD-1. PD-1 possesses immunoreceptor tyrosine-based inhibition motifs/switch motifs [22]. However, Tim-3 does not contain these two motifs and is not likely to be functionally redundant with checkpoint receptors containing these motifs. Taken together, Tim-3 may be a unique and promising immune checkpoint receptor. There are a number of ongoing clinical trials evaluating the efficacy of anti-Tim-3 therapies, especially in combination with PD-1 [23].

Galectin-9 is the first identified ligand of Tim-3. The galectin-9/Tim-3 axis is an important negative regulator of CD8^+^ cytotoxic T cell function [22]. Tim-3 suppresses antitumor response via non-T cell mechanisms involving Tim-3/Tim-3 ligand interactions, such as the interaction between tumor-associated dendritic cell-derived Tim-3 and HMGB1. In this study, we focused on the ligand galectin-9 and performed direct targeted deep sequencing of the galectin-9 gene. Our results revealed three missense mutations in six patients with DLBCL with a mutation rate of 3.2%. The mutation rate of galectin-9 varied between 0 and 2.5% for different cancers according to the COSMIC database. Notably, our study displayed that patients with DLBCL harboured a higher frequency of mutations in galectin-9 as compared to those with other cancers. The nucleotide substitutions identified were significantly different from those reported in patients with haematopoietic and lymphoid malignancy according to the COSMIC database. Using two prediction algorithms, the c.905G > A mutation was predicted to have a significant effect on the structure and/or function of the protein. Some amino acid residues are important for receptor-ligand interaction, and mutations at these sites enhance or interfere with protein-protein interaction [24]. Hence, it is vital to determine the genetic landscape of galectin-9 and comprehensively understand the role of Tim-3 signalling in the development of DLBCL.

A previous study has demonstrated that Tim-3 is expressed more on peripheral CD4^+^ and CD8^+^ T cells in patients with DLBCL [25]. However, tumor tissues provided better evidence for the tumor microenvironment as compared to peripheral blood. Chen et al. reported the correlation between high expression of Tim-3 on tumor cells and poor survival of patients with DLBCL [26]. Tim-3 is primarily expressed on TILs and impairs antitumor immune response by regulating the function of TILs. Although Tim-3 is positive on tumor cells in a minor population of patients with DLBCL, it is important to determine the prognostic significance of Tim-3 levels on TILs. Furthermore, the density and distribution of TILs have been shown to associate with the efficacy of immunotherapy. Based on the status of TILs, the tumor immune microenvironment can be classified into three phenotypes, namely, the immune-inflamed, immune-excluded, and immune-desert phenotypes [16]. The immune-inflamed phenotype is characterised by the presence of CD4^+^ and CD8^+^ T cells in the tumor parenchyma. Patients with this phenotype are most likely to benefit from immunotherapy. CD8^+^ T cells are key players that directly kill tumor cells and maintain immune surveillance [27]. In this study, we determined the expression of Tim-3 on TILs and CD8^+^ T cells and their association with clinical factors. To precisely define positive expression on cells, the absolute cell numbers per mm^2^ positive for Tim-3 and CD8 expression were calculated using a professional computer-assisted platform. DLBCL patients with Tim-3^+^ TILs showed poor survival compared with those with Tim-3^−^ TILs. The negative impact of Tim-3 on the prognosis has been demonstrated in other solid tumors, such as non-small-cell lung cancer [28], gastric cancer [29], and oesophageal squamous cell carcinoma [30]. Taken together, data from this study and others revealed an important role of Tim-3 in immune escape and poor prognosis of patients with solid and haematologic malignancies, suggesting that Tim-3 may be an important and promising target for immunotherapy.

Despite the cytolytic activity against tumors, CD8^+^ T cells lose this functional potential in the presence of chronic antigens, thereby generating exhausted T cells. PD-1 is a surrogate marker for T cell exhaustion [12]. However, a recent study has shown that exhaustion of CD8^+^ T cells also occurs in the absence of PD-1 [31], suggesting that other immune checkpoints also mediate CD8^+^ T cell exhaustion. Clinical responses to PD-1/PD-L1 inhibitors have not been satisfactory in patients with DLBCL; the rate of objective response is only 10% or less [3]. Hence, the indiscriminate use of PD-1 as a marker for exhausted CD8^+^ T cells in patients with DLBCL is inappropriate. Herein, we defined Tim-3 expression on CD8^+^ T cells as a novel subtype of exhausted T cells, namely, the exhausted Tim-3^+^ CD8^+^ T cells. Subsequently, we investigated the clinical significance of exhausted Tim-3^+^ CD8^+^ T cells in DLBCL. Tumors exhibit three different immune infiltration profiles. Immune cells with the “immune-excluded phenotype” are retained in the stroma and are unable to penetrate the tumor parenchyma. However, DLBCL is characterised by the diffuse proliferation of large neoplastic B lymphoid cells with no clear distinction between parenchyma and stroma [32]. Patients with the “immune-desert phenotype” usually exhibit low CD8^+^ T cell infiltration. Thus, we were unable to analyse the exhausted Tim-3^+^ CD8^+^ T cells and nonexhausted Tim-3^−^ CD8^+^ T cells in the above two phenotypes. Here, we focused on cells with the “immune-inflamed phenotype.” However, the phenotype has not been quantitatively analysed. To the best of our knowledge, this is the first report on the definition and quantification of CD8-expressing T cells that further investigates the role of exhausted CD8^+^ T cells in this phenotype. The exhausted Tim-3^+^ CD8^+^ T cells correlated with an unfavourable prognosis of DLBCL. We speculate that patients with this phenotype are more likely to be the ideal candidates for anti-Tim-3 therapy.

In conclusion, this study provides important genetic data for galectin-9 in patients with DLBCL. Future detailed mechanistic exploration is, however, required. The expression of Tim-3 on TILs and exhausted Tim-3^+^ CD8^+^ T cells was found to be associated with the poor survival of patients with DLBCL. Our data supported that Tim-3 mediated immunosuppression via the activity of cytotoxic CD8^+^ T cells and other tumor-infiltrating lymphocytes. This will help develop novel strategies in targeting Tim-3, perhaps even in combination with other immune checkpoint antibodies, for treatment of patients with DLBCL.

## Figures and Tables

**Table 1:** Association between Tim-3 expression on TILs and clinicopathologic parameters.

Parameters	*N* (%)	Tim-3^−^ TILs	Tim-3^+^ TILs	*p* value
Sex				
Female	67 (50)	30 (44.8)	38 (56.7)	0.167
Male	67 (50)	38 (55.2)	29 (43.3)	
Age (years)				
≤60	82 (67.7)	38 (56.7)	44 (65.7)	0.287
>60	52 (32.3)	29 (43.3)	23 (34.3)	
B symptom				
Yes	107 (86.2)	55 (82.1)	52 (77.6)	0.518
No	27 (13.8)	12 (17.9)	15 (22.4)	
Clinical stage				
I-II	64 (47.8)	38 (56.7)	26 (38.8)	0.038
III-IV	70 (52.2)	29 (43.3)	41 (61.2)	
IPI scores				
0-2	95 (73.8)	53 (79.1)	42 (62.7)	0.036
3-5	39 (26.2)	14 (20.9)	25 (37.3)	
LDH				
Normal	82 (55.4)	47 (70.1)	35 (52.2)	0.033
High	52 (44.6)	20 (29.9)	32 (47.8)	
Subtypes				
GCB	44 (32.8)	22 (32.8)	22 (32.8)	1.000
Non-GCB	76 (56.7)	38 (56.7)	38 (56.7)	
Unclassified	14 (10.4)	7 (10.4)	7 (10.4)	

## Data Availability

The data used to support the findings of this study are available from the corresponding authors upon request.

## References

[1] Li S., Young K. H., Medeiros L. J. (2018). Diffuse large B-cell lymphoma. *Pathology*.

[2] Gangadhar T. C., Salama A. K. (2015). Clinical applications of PD-1-based therapy: a focus on pembrolizumab (MK-3475) in the management of melanoma and other tumor types. *Oncotargets and Therapy*.

[3] Ansell S. M., Minnema M. C., Johnson P. (2019). Nivolumab for relapsed/refractory diffuse large B-cell lymphoma in patients ineligible for or having failed autologous transplantation: a single-arm, phase II study. *Journal of Clinical Oncology*.

[4] Burugu S., Dancsok A. R., Nielsen T. O. (2018). Emerging targets in cancer immunotherapy. *Seminars in Cancer Biology*.

[5] Sánchez-Fueyo A., Tian J., Picarella D. (2003). TIM-3 inhibits T helper type 1-mediated auto- and alloimmune responses and promotes immunological tolerance. *Nature Immunology*.

[6] Fourcade J., Sun Z., Benallaoua M. (2010). Upregulation of TIM-3 and PD-1 expression is associated with tumor antigen-specific CD8^+^ T cell dysfunction in melanoma patients. *The Journal of Experimental Medicine*.

[7] Baitsch L., Baumgaertner P., Devêvre E. (2011). Exhaustion of tumor-specific CD8^+^ T cells in metastases from melanoma patients. *The Journal of Clinical Investigation*.

[8] Sakuishi K., Apetoh L., Sullivan J. M., Blazar B. R., Kuchroo V. K., Anderson A. C. (2010). Targeting Tim-3 and PD-1 pathways to reverse T cell exhaustion and restore anti-tumor immunity. *The Journal of Experimental Medicine*.

[9] Zhou Q., Munger M. E., Veenstra R. G. (2011). Coexpression of Tim-3 and PD-1 identifies a CD8^+^ T-cell exhaustion phenotype in mice with disseminated acute myelogenous leukemia. *Blood*.

[10] Du W., Yang M., Turner A. (2017). TIM-3 as a target for cancer immunotherapy and mechanisms of action. *International Journal of Molecular Sciences*.

[11] Ngiow S. F., von Scheidt B., Akiba H., Yagita H., Teng M. W. L., Smyth M. J. (2011). Anti-TIM3 antibody promotes T cell IFN-*γ*-mediated antitumor immunity and suppresses established tumors. *Cancer Research*.

[12] Ahmadzadeh M., Johnson L. A., Heemskerk B. (2009). Tumor antigen-specific CD8 T cells infiltrating the tumor express high levels of PD-1 and are functionally impaired. *Blood*.

[13] Wilker P. R., Sedy J. R., Grigura V., Murphy T. L., Murphy K. M. (2007). Evidence for carbohydrate recognition and homotypic and heterotypic binding by the TIM family. *International Immunology*.

[14] Cibulskis K., Lawrence M. S., Carter S. L. (2013). Sensitive detection of somatic point mutations in impure and heterogeneous cancer samples. *Nature Biotechnology*.

[15] Wang X., Zhang T., Song Z. (2019). Tumor CD73/A2aR adenosine immunosuppressive axis and tumor-infiltrating lymphocytes in diffuse large B-cell lymphoma: correlations with clinicopathological characteristics and clinical outcome. *International Journal of Cancer*.

[16] Chen D. S., Mellman I. (2017). Elements of cancer immunity and the cancer-immune set point. *Nature*.

[17] Shi Y., Deng L., Song Y. (2018). CD3^+^/CD8^+^ T-cell density and tumoral PD-L1 predict survival irrespective of rituximab treatment in Chinese diffuse large B-cell lymphoma patients. *International Journal of Hematology*.

[18] Rajnai H., Heyning F. H., Koens L. (2014). The density of CD8^+^ T-cell infiltration and expression of BCL2 predicts outcome of primary diffuse large B-cell lymphoma of bone. *Virchows Archiv*.

[19] Hamid O., Robert C., Daud A. (2013). Safety and tumor responses with lambrolizumab (anti-PD-1) in melanoma. *The New England Journal of Medicine*.

[20] Gao X., Zhu Y., Li G. (2012). TIM-3 expression characterizes regulatory T cells in tumor tissues and is associated with lung cancer progression. *PLoS One*.

[21] Yang Z. Z., Grote D. M., Ziesmer S. C. (2012). IL-12 upregulates TIM-3 expression and induces T cell exhaustion in patients with follicular B cell non-Hodgkin lymphoma. *The Journal of Clinical Investigation*.

[22] Rangachari M., Zhu C., Sakuishi K. (2012). Bat3 promotes T cell responses and autoimmunity by repressing Tim-3-mediated cell death and exhaustion. *Nature Medicine*.

[23] Hahn A. W., Gill D. M., Pal S. K., Agarwal N. (2017). The future of immune checkpoint cancer therapy after PD-1 and CTLA-4. *Immunotherapy*.

[24] Lim H., Chun J., Jin X., Kim J., Yoon J. H., No K. T. (2019). Investigation of protein-protein interactions and hot spot region between PD-1 and PD-L1 by fragment molecular orbital method. *Scientific Reports*.

[25] Xiao T., Zhang L., Chen L., Liu G., Feng Z., Gao L. (2014). Tim-3 expression is increased on peripheral T cells from diffuse large B cell lymphoma. *Tumour Biology*.

[26] Chen B. J., Dashnamoorthy R., Galera P. (2019). The immune checkpoint molecules PD-1, PD-L1, TIM-3 and LAG-3 in diffuse large B-cell lymphoma. *Oncotarget*.

[27] Ribas A., Shin D. S., Zaretsky J. (2016). PD-1 blockade expands intratumoral memory T cells. *Cancer Immunology Research*.

[28] Zhuang X., Zhang X., Xia X. (2012). Ectopic expression of TIM-3 in lung cancers: a potential independent prognostic factor for patients with NSCLC. *American Journal of Clinical Pathology*.

[29] Jiang J., Jin M. S., Kong F. (2013). Decreased galectin-9 and increased Tim-3 expression are related to poor prognosis in gastric cancer. *PLoS One*.

[30] Hong M. H., Shin S. J., Shin S. K. (2019). High CD3 and ICOS and low TIM-3 expression predict favourable survival in resected oesophageal squamous cell carcinoma. *Scientific Reports*.

[31] Odorizzi P. M., Pauken K. E., Paley M. A., Sharpe A., Wherry E. J. (2015). Genetic absence of PD-1 promotes accumulation of terminally differentiated exhausted CD8^+^ T cells. *The Journal of Experimental Medicine*.

[32] Kiyasu J., Miyoshi H., Hirata A. (2015). Expression of programmed cell death ligand 1 is associated with poor overall survival in patients with diffuse large B-cell lymphoma. *Blood*.

