# Corrigendum to “Genetic Mutations of Tim-3 Ligand and Exhausted Tim-3^+^ CD8^+^ T Cells and Survival in Diffuse Large B Cell Lymphoma”

**DOI:** 10.1155/2021/4972043

**Published:** 2021-01-30

**Authors:** Tingting Zhang, Tianyuan Ren, Zheng Song, Jing Zhao, Lei Jiao, Zhenzhen Zhang, Jin He, Xianming Liu, Lihua Qiu, Lanfang Li, Shiyong Zhou, Bin Meng, Qiongli Zhai, Xiubao Ren, Zhengzi Qian, Xianhuo Wang, Huilai Zhang

**Affiliations:** ^1^Department of Lymphoma, Tianjin Medical University Cancer Institute and Hospital, National Clinical Research Center of Cancer, Key Laboratory of Cancer Prevention and Therapy, Tianjin's Clinical Research Center for Cancer, The Sino-US Center for Lymphoma and Leukemia Research, Tianjin, China; ^2^Panovue Biological Technology Co., Ltd., Beijing, China; ^3^Marvel Medical Laboratory, Tianjin Marvelbio Technology Co. Ltd., Tianjin, China; ^4^Department of Pathology, Tianjin Medical University Cancer Institute and Hospital, Tianjin, China; ^5^Department of Immunology/Biotherapy, Tianjin Medical University Cancer Institute and Hospital, Tianjin, China

In the article titled “Genetic Mutations of Tim-3 Ligand and Exhausted Tim-3^+^ CD8^+^ T Cells and Survival in Diffuse Large B Cell Lymphoma” [1], the survival data for the Tim-3^+^ TIL and Tim-3^−^ TIL groups was mistakenly reversed by the authors during manuscript preparation. The following sections should be corrected.

- The abstract section should be corrected from:

“Multiplexed immunofluorescence revealed that patients with Tim-3-expressing tumor-infiltrating lymphocytes (Tim-3^+^ TILs) exhibited poor outcomes than those with Tim-3^−^ TILs (*p* = 0.041). The median survival times of these patients were 65.0 (95% confidence interval (CI): 71.2–88.6) and 79.9 months (95% CI: 54.4–75.6), respectively.”

To:

“Multiplexed immunofluorescence revealed that patients with Tim-3-expressing tumor-infiltrating lymphocytes (Tim-3^+^ TILs) exhibited poor outcomes than those with Tim-3^−^ TILs (*p* = 0.041). The median survival times of these patients were 65.0 (95% confidence interval (CI): 54.4–75.6) and 79.9 months (95% CI: 71.2–88.6), respectively.”

(ii) Section 3.4, “Prognostic value of Tim-3^+^ TILs and exhausted Tim-3^+^ CD8^+^ T cells in DLBCL,” should be corrected from:

“The median survival times for patients with Tim-3^+^ TILs and Tim-3^−^ TILs were 65.0 (95% confidence interval (CI): 71.2–88.6) and 79.9 months (95% CI: 54.4–75.6), respectively.”

To:

“The median survival times for patients with Tim-3^+^ TILs and Tim-3^−^ TILs were 65.0 (95% confidence interval (CI): 54.4–75.6) and 79.9 months (95% CI: 71.2–88.6), respectively.”
